# Multivariate Time Series Anomaly Detection Based on Inverted Transformer with Multivariate Memory Gate

**DOI:** 10.3390/e27090939

**Published:** 2025-09-08

**Authors:** Yuan Ma, Weiwei Liu, Changming Xu, Luyi Bai, Ende Zhang, Junwei Wang

**Affiliations:** 1 The Center of National Railway Intelligent Transportation System Engineering and Technology, China Academy of Railway Sciences Corporation Limited, Beijing 100081, China; 13910233118@139.com; 2 School of Computer Science and Technology, Northeastern University at Qinhuangdao, Qinhuangdao 066004, China; 2372452@stu.neu.edu.cn (W.L.); changmingxu@neuq.edu.cn (C.X.); wangjunwei@neuq.edu.cn (J.W.); 3 School of Computer Science and Engineering, Northeastern University, Shenyang 110819, China; zed@cc.neu.edu.cn

**Keywords:** time series, anomaly detection, transformer, deep learning, unsupervised learning

## Abstract

In the industrial IoT, it is vital to detect anomalies in multivariate time series, yet it faces numerous challenges, including highly imbalanced datasets, complex and high-dimensional data, and large disparities across variables. Despite the recent surge in proposals for deep learning-based methods, these approaches typically treat the multivariate data at each point in time as a unique token, weakening the personalized features and dependency relationships between variables. As a result, their performance tends to degrade under highly imbalanced conditions, and reconstruction-based models are prone to overfitting abnormal patterns, leading to excessive reconstruction of anomalous inputs. In this paper, we propose ITMMG, an inverted Transformer with a multivariate memory gate. ITMMG employs an inverted token embedding strategy and multivariate memory to capture deep dependencies among variables and the normal patterns of individual variables. The experimental results obtained demonstrate that the proposed method exhibits superior performance in terms of detection accuracy and robustness compared with existing baseline methods across a range of standard time series anomaly detection datasets. This significantly reduces the probability of misclassifying anomalous samples during reconstruction.

## 1. Introduction

Time series data has extensive applications across a wide range of disciplines, such as financial markets, industrial manufacturing, intelligent transportation, medical monitoring, and environmental monitoring [1]. Anomaly detection is a pivotal component of time series analysis, which pertains to the identification of points or intervals in the time series data that deviate from the norm expected features to detect potential fault risks [2]. With the rapid development of the IoT in industrial environments, an increasing number of sensors are being deployed for machine condition monitoring, generating vast amounts of multivariate time series data.

However, time series anomaly detection is complex and challenging, with diverse data, large scale, and various anomaly types, and there is a serious imbalance between normal and abnormal data. There are many normal samples and few abnormal samples, which often makes rare anomaly signals concealed by a large quantity of normal data; thus, it is difficult to accurately mark all historical data. In order to meet this challenge, unsupervised anomaly detection methods for multivariate time series have gradually become a focal point for research. Anomaly detection methods in the classical tradition, based on statistics and machine learning methods [3] (such as isolated forest [4], support vector machine [5], and local outlier factor [6]), perform well in dealing with low-dimensional and univariate time series. However, when faced with complex and high-dimensional time series, these methods are usually difficult to capture time dependence and variable correlation effectively and are easily affected by data noise.

Therefore, more research turns to using a deep learning model to build a more complex and flexible anomaly detection model. Advances in the field of artificial intelligence technology have precipitated a series of significant developments in the method of deep learning [7]. Reconstruction-based techniques constitute a fundamental paradigm in multivariate time series anomaly detection, leveraging the ability to faithfully reconstruct normal patterns while exhibiting significant reconstruction deviations for anomalous inputs. Because of the strong interpretability of reconfiguration-based methods, some research works have performed anomaly detection tasks in a reconfiguration-based manner. For example, TranAD [8] combines Transformer with Generative Adversarial Networks (GANs) to detect anomalies through a reconfiguration-based encoder–decoder architecture. Anomaly Transformer [9] introduces an innovative Anomaly Attention mechanism, which combines correlation differences and refactoring to distinguish outliers. Wang et al. propose a frequency-oriented VAE framework revisiting anomaly detection from spectral perspectives. Because the time series data often presents an extreme imbalance between typical and atypical samples in the actual scene, the reconstruction method has certain challenges in modeling.

In constructing time series embeddings, the conventional method represents all variables at each time point as a monolithic entity (a single token), which ignores the individualized characteristics and diversity among variables and has shortcomings in accurately capturing the complex associations of different variables [10]. Different points of the same time step often represent the physical quantities measured by different sensors, and these variables may have a unique correlation. However, embedding these measured values directly into a single word containing multivariate data will weaken this correlation and cover up the characteristics of individual variables. In addition, the markers formed by a single time step are highly localized in the receptive field, which not only limits the model’s capability of identifying cross-variables and long-term dependencies, but is also easily affected by misaligned events at time points, which further weakens the effectiveness of the model. Existing reconstruction-based methods are prone to over-generalization [11,12]. Because the encoder may extract abnormal features or the decoder has too strong reconstruction ability, the abnormal input may be accurately reconstructed. Such methods usually assume that the characteristics of all time steps can be represented by a unified reconstruction pattern, ignoring the differences in the relationship between different variables. In fact, each variable often presents a unique normal pattern, and the characteristics of different variables are easily confused, which affects the precision of anomaly recognition.

To tackle the identified challenges, we propose an inverted Transformer framework combined with improved multi-memory gating (ITMMG) for the detection of abnormal patterns in multivariate temporal data. ITMMG uses an inverted token encoding strategy. Specifically, the inverted token strategy encodes the time step data in the window of each variable into a token independently, which makes the model flexibly deal with complex multidimensional time series data. In view of the unique normal pattern of different variables, this paper proposes a new multivariate memory gate, which sets independent memory units for each variable. Each variable learns its normal pattern through its unique memory item. It can better adapt to the variable differences in multivariate time series in order to better deal with the feature confusion caused by the dependent variable differences and more accurately identify instances with notable discrepancies compared to normal patterns during anomaly detection.

While the proposed framework may exhibit reduced sensitivity to gradual trend anomalies—a subtype of collective anomalies characterized by slow-evolving deviations—it maintains robust overall effectiveness on datasets encompassing such patterns. Empirical validation of SWaT and NIPS-TS-SWAN (where trend anomalies challenge detection models) demonstrates competitive F1 scores of 70–90%, indicating practical utility despite theoretical limitations. This balanced performance across diverse anomaly types underscores our model’s viability as a general-purpose detection solution.

The main contributions of this paper can be summarized as follows:

- We propose a multi-memory gated Transformer framework based on inverted embedding. Through fine-grained modeling of complex interactions and dependencies among variables, global correlation learning is strengthened;
- We propose an improved multi-memory unit and self-supervised modeling mechanism, which can capture the specific normal pattern of each variable and adaptively adjust the memory state;
- The most advanced performance is achieved in a large number of experiments on four widely used benchmark datasets.

The remainder of this paper is structured as follows: The Section 2 of this text is dedicated to an examination of the literature surrounding the subject. Section 3 provides a detailed description of the problem and of the proposed ITMMG method. In Section 4, we describe the experimental methodology, summarize the evaluation findings, and conduct an ablation study. The paper’s final conclusions are drawn in Section 5.

## 2. Related Work

### 2.1. Detection of Time Series Anomalies

Finding anomalous samples that differ from the majority of data that are frequently linked to problems like equipment failures or structural flaws is the main goal of research in the subject of time series anomaly detection. Univariate anomaly detection focuses on abnormal behaviors of a single parameter. In contrast, multivariate anomalies involve joint abnormal behavior across multiple parameters, which makes the model construction more complicated because of the complex correlation between sequences.

Depending on the data labels, the current anomaly detection algorithms can be classified as either supervised or unsupervised. Because it is difficult to obtain data labels in practical applications, unsupervised methods are more common. Algorithms can also be classified according to classic machine learning and deep learning. The former includes a linear model, distance method and probability density estimation. For example, the Gaussian mixture model and deep neural network are combined in DAGMM [13]. With the development of neural networks, methods based on deep learning also appear in this field, such as employing deep learning models like graph neural networks (GNN [14]). LSTM-VAE [15] is a variational self-encoder model, replacing the traditional feedforward network with LSTM, and Omnianomaly [16] uses random cyclic neural networks, similar to LSTM variational automatic encoder and planar normalized flow, to generate reconstruction probability. Graph Learning with Transformer for Anomaly detection (GTA [17]) learns the link between several IoT sensors by using graph structure. TimeMixer [18] and HDMixer [19] recently demonstrated the efficacy of multiscale mixing in forecasting. In addition, a lot of work uses the method based on generating a confrontation network (GAN) [20,21,22] and the method based on deep reinforcement learning (DRL) [23]. Reconstruction techniques are the foundation of the majority of these deep learning models. Excellent accuracy in detection is achievable due to deep learning’s potent representation learning capability. Researchers have recently suggested a technique that uses self-supervised learning to increase the generalization capacity of unsupervised detection of anomalies. Recent studies have extended these methods to critical infrastructure domains, such as railway operations leveraging edge computing [24] and structural health monitoring systems for bridges [25].

### 2.2. Embedding and Tokenization of Time Series Data

Embedding technology is fundamentally a mathematical framework for mapping structured objects into low-dimensional continuous vector spaces [26]. Initially achieving breakthroughs in natural language processing, it successfully transformed discrete symbols (e.g., words, phrases) into dense vector representations to capture semantic relationships. With advancements in deep learning, the applicability of embeddings has significantly expanded to continuous data domains, including patch embedding representations in computer vision [27], node and edge embeddings in graph neural networks [28], speech recognition [29], etc.

While established segmentation/tokenization algorithms underpin effective word embeddings for natural language, the analogous partitioning of continuous objects—such as images, audio, and time series data—does not readily present equally simple and effective solutions, making the segmentation of time series particularly worthy of attention. The pursuit of versatile representations for heterogeneous time series data continues to pose significant hurdles, as highlighted in contemporary literature reviews [30]. This persistent challenge substantiates our specialized approach toward anomaly-centric feature learning, diverging from universal representation paradigms.

In multivariate time series modeling, data exhibits dual dimensions: temporal steps and variables. Conventional Transformer architectures typically adopt the temporal tokenization paradigm [31], where all variables at the same timestep are encoded into a single token vector. This representation treats timesteps as fundamental units, modeling temporal dependencies through attention mechanisms. In contrast, iTransformer innovatively proposes variable tokenization [10], treating entire temporal sequences of individual variables as independent tokens to achieve entity-centric embedding construction, explicit inter-variable interaction modeling, and preservation of variable-specific patterns. This paradigm shift demonstrates embedding techniques’ adaptability to domain-specific inductive biases through aligned vectorized representations, providing novel multivariate time series feature-learning perspectives with validated superiority. Unlike T-Rep’s time embeddings [32] that explicitly encode temporal positions, our inverted embedding discards absolute timing to prioritize cross-variable dependencies.

### 2.3. Transformer-Based Time Series Analysis

Transformer [33] has been successfully deployed in the area of natural language processing for the first time. Thanks to its powerful ability in sequential data processing, Transformer has also achieved considerable success in a variety of domains, including but not limited to audio processing, natural language processing, and computer vision [27,34]. The efficacy of the self-attention mechanism in addressing the long-distance dependence of time series data has led to its extensive utilization in the domain of time series analysis in recent years. Anomaly Transformer [9], TranAD [8], AnomalyBert [35] and so on have extensively explored the application of Transformer in anomaly detection methods. MEMTO [36] uses a Transformer encoder as feature extraction and proposes a module including memory gating to solve the detection error based on the reconstructed model. These modeling methods usually embed multivariate data through time steps. Although this method can capture the dependencies of time dimensions, it is insufficient for modeling the feature association between variables. In contrast, this paper uses an inverted embedding strategy to enhance the capture ability of complex interactions between variables and combines the method of multivariate memory units to capture the personalized features of variables more accurately.

## 3. Methods

### 3.1. Definition of the Problem

The dataset is separated into training and test sets based on the unsupervised time series anomaly detection task. The training set contains no anomaly labels. During training, the model learns the typical data patterns solely by leveraging intrinsic data associations, such as temporal dependencies and inter-variable relationships. The test set contains labels to evaluate the anomaly scores inferred by the model and assess its detection performance.

We formalize the core notation for multivariate temporal segmentation and anomaly detection mechanics to ensure mathematical clarity throughout this work.

**Definition** **1** **(Temporal Partitioning).** *Given a multivariate time series* $S\in R^{N\times T_{S}}$ *with N* *variables and $T_{S}$* *timesteps, we partition S into L* *contiguous windows ${\left\{X^{\left(l\right)}\right\}}_{l=1}^{L}\subset \;S$, where each window spans exactly T timesteps, such that $card\left(\overset{L}{\underset{l=1}{⋃}}X^{\left(l\right)}\right)=T_{s}$,* *where $card\left(\;\right)$* *denotes the count of distinct timesteps.*

**Definition** **2** **(Anomaly Detection Criterion).** *Given* $S\in R^{N\times T_{S}}$*, the anomaly detection task reduces to identifying windows $X^{\left(l\right)}$* *(from Definition 1), where the anomaly score ϕ($X^{\left(l\right)}$) exceeds a predefined threshold δ. The set of anomalous windows is*

(1) $$\Phi \left(S\right)=\left\{X^{\left(l\right)}\;\right|\;\phi (X^{\left(l\right)})>\delta ,\;1\leq l\leq L\}$$

*A time series S is flagged as anomalous if *Φ(S) *is non-empty.*

This window-based formulation enables localized anomaly detection while maintaining computational tractability. Its efficacy stems from capturing local temporal dependencies, though the global context beyond window boundaries may be attenuated. The original multivariate time series data is partitioned into fixed-length, contiguous windows $X\in R^{T\times N}$. The collection of these windows {X} forms the dataset for both training and test. For each window *X*, our model aims to learn its underlying normal patterns.

### 3.2. Model Overview

**Figure 1:**
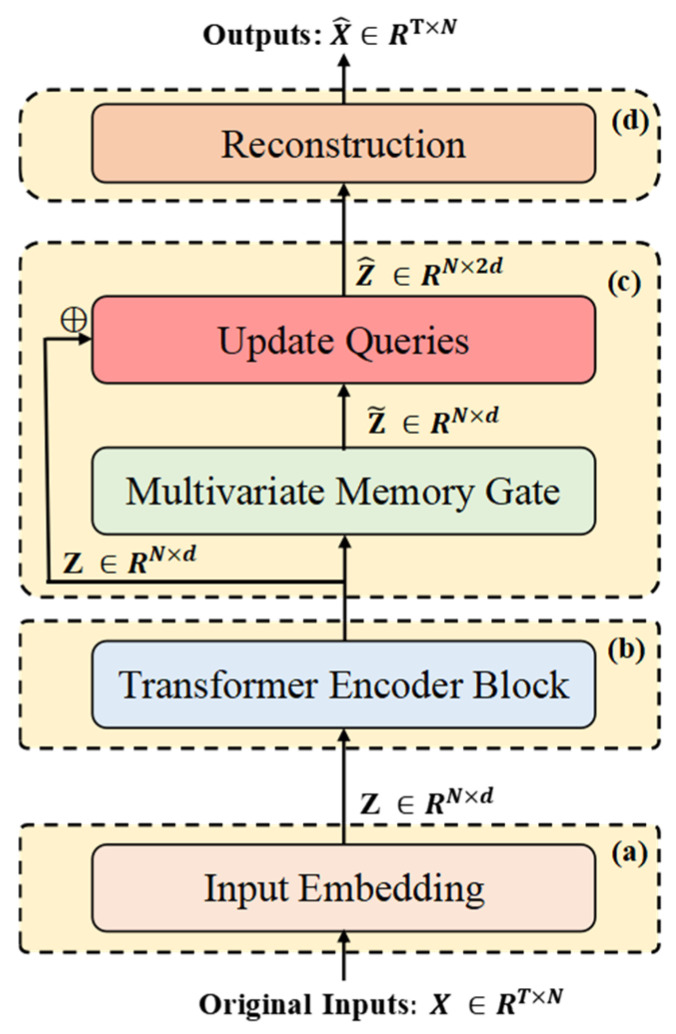
The architecture of ITMMG. (**a**) Raw data of different variables are used as markers and independently embedded to obtain different representations. (**b**) Transformer encoder as the backbone for feature extraction. (**c**) The multivariate memory re-expresses the latent states output by the Transformer encoder in its learned latent state space. (**d**) The reconstruction module, as a simple decoder, is incapable of effectively reconstructing features of anomalous patterns, thus amplifying reconstruction errors for anomalous data.

Figure 1 illustrates the overall architecture of ITMMG. The framework comprises four core components: (1) multi-dimensional time series embedding, (2) Transformer encoder, (3) multivariate memory gate, and (4) reconstruction decoder.

Input windows *X* undergo tokenization to generate variable-oriented representations. This transformation enhances the capture of intra-window dependencies, enabling precise characterization of normal behavioral patterns within individual windows—essential for window-level anomaly detection during inference. The Transformer encoder performs feature extraction, capturing horizontal dependencies between variables and encoding the input data within the time series window into latent vector representations. Within the multi-query gating module, (1) dedicated memory modules are assigned per variable, (2) latent representations are decomposed into asynchronous variable-specific vectors, (3) these vectors update corresponding variable memory modules, (4) and the updated representations fuse with original latent vectors. Final fused vectors enter the reconstruction decoder to recover the original subsequence. Anomaly scores derive jointly from reconstruction error and latent spatial bias.

### 3.3. Input Embedding

**Figure 2:**
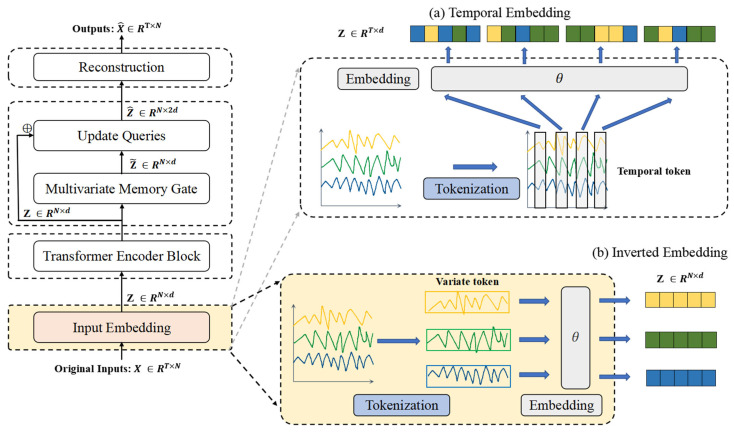
The input embedding. (**a**) Temporal embedding: Adopts temporal tokenization (aggregating all variables per timestep into a single token) to construct embeddings, primarily excelling in directly modeling temporal dependencies. (**b**) Inverted embedding: Utilizes variable tokenization (treating each variable’s full time series as an independent token) with dimension swapping to formulate embeddings, whose key strength lies in explicitly capturing cross-variable interactions.

In multivariate time series modeling, data exhibits dual dimensions: temporal steps and variables. This work employs an inverted embedding strategy (visualized in Figure 2b), where feature dimensions—rather than temporal steps—serve as the primary embedding axis.

This approach projects multivariate observations at each timestep into a latent space $Z^{0}=\{h_{1},\ldots ,h_{N}\}\in R^{N\times d}$, where *d* denotes embedding dimensionality. This technique fundamentally diverges from sequential tokenization by explicitly prioritizing cross-variable dependency modeling over temporal pattern extraction. Through dimension-swapping operations, it transforms each variable’s full time series into an independent latent representation, thereby directly capturing synchronous interactions between channels. Consequently, it circumvents the computational redundancy of temporal-step embeddings while enhancing sensitivity to synchronous anomalies across channels—a critical advantage for high-dimensional industrial sensors like the dataset SWaT’s 51-variable system.

### 3.4. Feature Extraction Encoder

The Transformer encoder is used to perform deep feature extraction on the token set of time series, which is dominated by variables. The main goal of this component is to reveal the horizontal relationships and dynamic features between multi-dimensional time series variables. This enhances the model’s capacity to capture the complex interactions between multiple variables.

**Figure 3:**
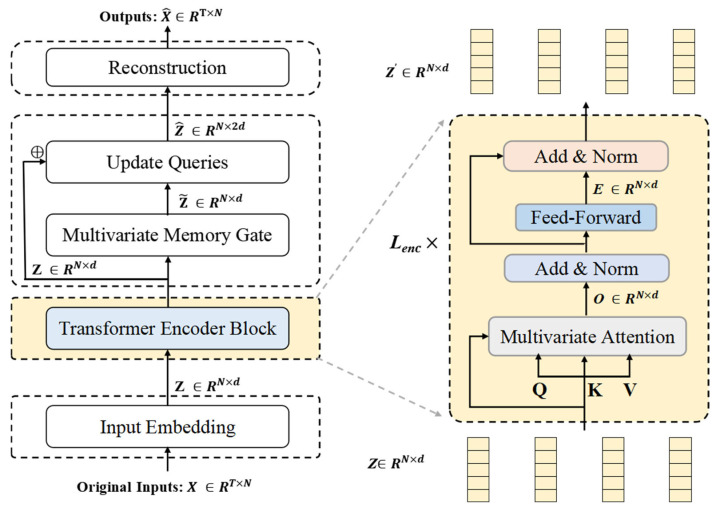
The structure of the Transformer encoder block. The encoder employs *L_enc_* stacked Transformer layers, with the internal structure of each layer illustrated in the right panel of the figure.

**Figure 4:**
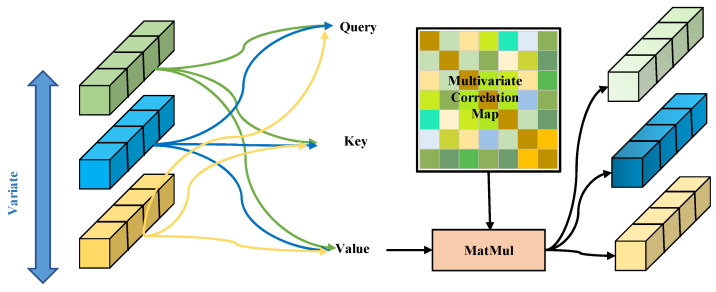
Multivariate attention. Multiple variables are embedded using multi-head self-attention to capture their complex interdependencies.

The structural composition of the Transformer encoder block is presented in Figure 3. A schematic diagram illustrating the multi-head attention mechanism under multivariate attention circumstances is provided in Figure 4. Note that the variable z in Figure 4 has dimensions *N* × *d*, indicating that each input vector represents an embedding of a specific variable rather than a temporal embedding. The inverted embedding applies a linear transformation to each subsequence: *Z* = $X^{T}W$, where $X\in R^{T\times N}$, $W\in R^{T\times d}$ is the projection matrix, output $Z\in R^{N\times d}$ preserves original timesteps, and no activation function or bias term is applied.

#### 3.4.1. Encoder Structure

The input data $X\in R^{T\times N}$ is embedded into a token set dominated by variables, forming the initial embedding representation $Z^{0}=\{h_{1},\ldots ,h_{N}\}\in R^{N\times d}$, where d is the embedding dimension for each variable. This embedding is then passed through the Transformer encoder consisting of *L_enc_* layers. Each layer contains multi-head self-attention, layer normalization, multi-head self-attention, and a feed-forward neural network.

In the multi-head self-attention mechanism, each head performs a linear projection Z to obtain $Q_{k}$, $K_{k}$, and $V_{k}$, where $d_{k}$ represents the projection dimension for each head, where 1≤k≤K. The attention outputs $O_{k}\in R^{N\times d_{k}}$ are computed using scaled dot-product attention:

(2) $$(O_{k}{)}^{T}=Softmax\left(\frac{Q_{k}K_{k}^{T}}{\sqrt{d_{k}}}\right)V_{k}$$

The encoder module also includes layer normalization and a feed-forward network with residual connections. It generates a representation for each variable $E\in R^{N\times d}$. In each layer of the encoder, due to the inverted sequence dimension of the input, each token represents the sequence of a variable along the time dimension. This allows the multi-head self-attention to effectively analyze the dependencies between variables and generate new representations for each variable. After the multi-head attention mechanism, the newly generated representations undergo nonlinear mapping and feature compression through the feed-forward network, enabling the model to extract complex representations from the original features to describe the time series. This process aids in identifying complex anomaly patterns that are difficult for linear models to capture.

#### 3.4.2. Reconstruction Decoder

Two fully connected layers make up the decoder. This design aims to balance the capabilities of the encoder and decoder, ensuring that the output accurately reflects the encoder’s processing results. This approach prevents an overly powerful decoder from accurately reconstructing random noise devoid of input data during the reconstruction process, which could introduce biases into the model during anomaly detection [36].

### 3.5. Multivariate Memory Module

To address the issues of overgeneralization in reconstruction-based models and variability among different variables in multivariate time series, where each variable may display specific normal pattern characteristics, we propose a solution. The differences in feature distribution and patterns across variables make it challenging for the model to learn and adapt to the distinct characteristics of each variable. Sharing a single memory module may cause the model to overlook certain variable patterns or confuse the features of different variables. Therefore, we introduce a novel multivariate gating module, which allows for the allocation of independent memory modules for each variable. The multivariate memory update requires calculating similarity across all memory units, which slightly reduces inference speed. This design allows the model to more effectively handle the variability among variables in multivariate time series.

**Figure 5:**
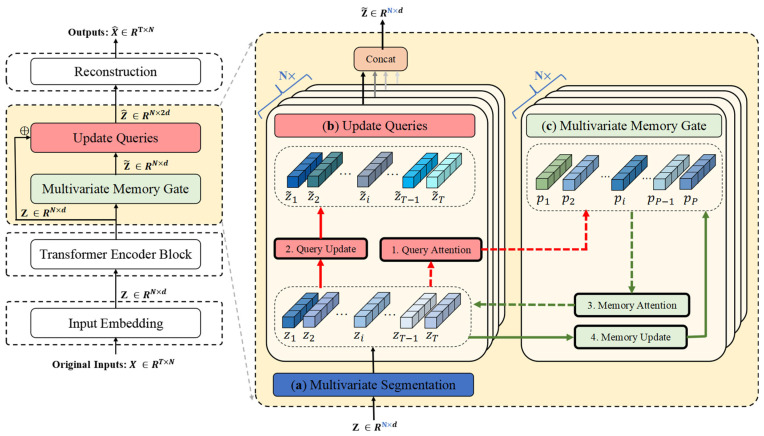
Multivariate memory module during training. (**a**) Multivariate segmentation: Each variable is assigned a dedicated memory module, thus preserving its unique pattern characteristics without cross-variable interference. (**b**) The Update Queries operation reconstructs the input embedding (denoted as query) by computing attention scores between the query and memory vectors, followed by weighted aggregation. (**c**) The multivariate memory gate leverages all queries to refresh memory vectors through attention-based weighting.

**Figure 6:**
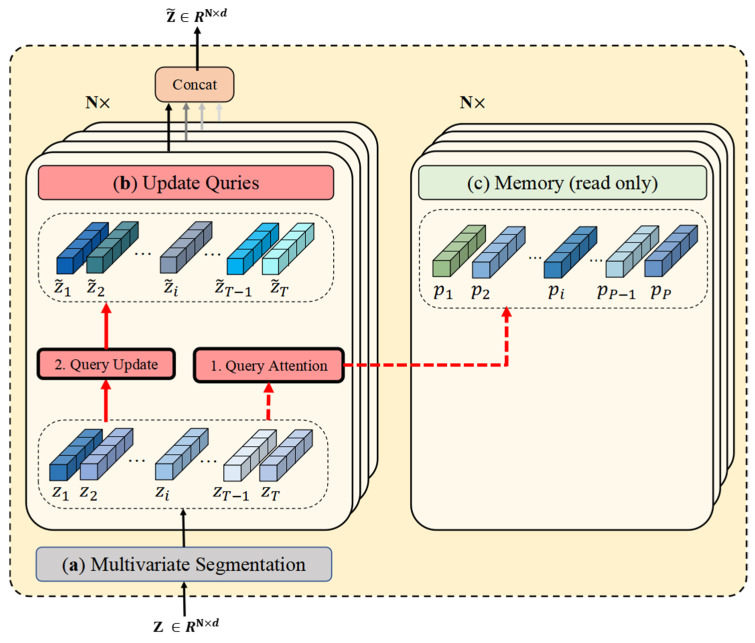
Multivariate memory module in inference mode. (**a**) Multivariate segmentation. (**b**) The Update Queries operation. (**c**) The multivariate memory gate (read only).

As delineated in Figure 5, the memory update via the multivariate gate is exclusively executed during the unsupervised learning phase. During inference, however, the memory module operates in the read-only mode solely for query processing. Figure 6 depicts the architecture of the multivariate memory module under this inference paradigm.

#### 3.5.1. Multivariate Memory Update

The memory units are denoted as $p_{i}^{j}$, where i∈[1, P] indexes the memory unit and j∈[1, N] indexes the variable. Additionally, P denotes the number of memory units per variable. The memory units are trained during the model training process to hold the typical data patterns for various variables. We train the model’s memory units such that each variable’s memory unit contains prototype features corresponding to the latent vectors of normal timesteps. The memory items for different variables are updated incrementally. The dot product is computed between each memory unit and the latent representation, followed by normalization using the Softmax function. Regarding the j-th variable’s data, the formula is as follows:

(3) $$v_{i,t}^{j}=\;\frac{\exp\left(\frac{\langle \;p_{i}^{j},\;z_{t}\rangle }{\tau }\right)}{\sum_{k=1}^{T}\exp\left(\frac{\langle \;p_{i}^{j},\;z_{k}\;\rangle }{\tau }\right)}$$

where τ represents the temperature hyperparameter. In the memory module, we utilize an update gate ψ to flexibly train each memory unit, making it suitable for diverse normal patterns. Based on the data, the model may then adaptively learn the update magnitude of each memory unit. Updating of the memory module unit occurs solely during training.

The following is the formula for the memory update:

(4) $$\psi =\;\sigma (U_{\psi \;}p_{i}^{j}+Q_{\psi }\sum_{t=1}^{T}v_{i,t}^{j}z_{t}\;)$$

(5) $$p_{i}^{j}=\left(1-\psi \right)\;⨀\;p_{i}^{j}+\psi \;\;⨀\;\sum_{t=1}^{T}v_{i,t}^{j}z_{t}\;$$

where $U_{\psi }$ and $Q_{\psi }$ represent the projections, and σ and ⊙ denote the sigmoid and element-wise multiplication.

#### 3.5.2. Latent Vector Update

In the latent vector update phase, an updated latent vector is generated for each variable. The query attention weight is defined and computed by applying the Softmax function to the dot product, as shown below:

(6) $$\;u_{i,t}^{j}=\;\frac{\exp\left(\frac{\langle \;p_{i}^{j},\;{\;z}_{t}\;\rangle }{\tau }\right)}{\sum_{w=1}^{P}\exp\left(\frac{\langle \;p_{w}^{j},\;\;z_{t}\;\rangle }{\tau }\right)}$$

Next, the retrieved memory item $p_{i}^{j}$ is weighted by $u_{i,t}^{j}$, and the corresponding latent vector ${\tilde{z}}_{t}$ is obtained.

(7) $${\tilde{z}}_{t}=\sum_{i^{\prime }=1}^{P}u_{i^{\prime },t}^{j}\;p_{i^{\prime }}^{j}$$

The retrieved latent vector from the memory unit for each variable is concatenated with the original latent vector, forming the updated final latent vector $\hat{Z_{t}}$. This updated latent vector $\hat{Z_{t}}$ serves as the input to the decoder. Since each variable memory unit continuously provides normal features, it results in significant bias in the abnormal data during reconstruction.

#### 3.5.3. Loss Function

In the training phase, we adopt reconstruction loss as the primary optimization objective and utilize entropy loss as an auxiliary regularization term to achieve sparsity in memory attention. The following is the definition of the total loss function, where λ is the hyperparameter to maintain balance.

(8) $${Loss}_{total}={Loss}_{recon}+\lambda {Loss}_{entr}$$

The reconstruction loss, which is defined as follows, guarantees that the model can reconstruct the input data precisely:

(9) $${Loss}_{recon}=\frac{1}{T}\sum_{t=1}^{T}{\left\|X_{t}-{\hat{X}}_{t}\right\|}_{2}^{2}$$

where $X_{t}$ denotes the input data and $\hat{X_{t}}$ denotes the reconstructed data. By minimizing $L_{recon}$, the model learns to achieve low reconstruction error for normal samples, while significantly increasing the reconstruction error for anomalous data, thus assisting in locating outlier samples in the dataset.

To enhance the attention to the memory units allocated for each variable, we introduce entropy loss regularization. This regularization term encourages the attention weights $u_{i,t}^{j}$ to exhibit a sparse distribution. The entropy loss is defined as

(10) $${Loss}_{entr}=\frac{1}{N}\sum_{j=1}^{N}\sum_{t=1}^{T}\sum_{i=1}^{P}-u_{i,t}^{j}\log\left(u_{i,t}^{j}\right)$$

The complete training process is shown in Algorithm 1.
**Algorithm 1** ITMMG Training Algorithm **Required:** Training data $X\in R^{T\times N}$; ITMMG model; number of epochs; block number *L_enc_*. Train encoder after first phase $01\text{:}\;Z^{rand}=f_{enc}(X)$ 02: P = K-$\mathrm{means}(Z^{rand}$) 03: n ← 1 04: **while** n ≤ epoch **do**: $05\text{:}\;Z^{0}$ = Embedding(*X*) 06:  for l = 1 to *L_enc_*: $07\text{:}\;Z^{l-1}$$\;=\;\mathrm{LayerNorm}(Z^{l-1}$$\;+\;\mathrm{Self-Attn}(Z^{l-1}$)) $08\text{:}\;Z^{l}$$\;=\;\mathrm{LayerNorm}(Z^{l-1}$$\;+\;\mathrm{Feed-Forward}(Z^{l-1}$)) 09:  **End for** 10:  **for** each variable in
$Z^{l}$ **do**: 11:    Compute attention scores 12:    Update memory p 13:  **End for** 14:  $\hat{Z^{L_{enc}}}=concat(\left[Z^{L_{enc}},\tilde{Z^{l}}\right],dim=1)$ 15:  $\hat{X}=f_{dec}(\hat{Z^{L_{enc}}})$ 16:  $\mathrm{Calculate}\;\mathrm{reconstruction}\;\mathrm{loss}\;{Loss}_{recon}$ 17:  $\mathrm{Calculate}\;{Loss}_{entr}=\;\sum_{j=1}^{N}{Loss}_{entr}^{j}$ 18:  $\mathrm{Calculate}\;{Loss}_{total}\;$ 19:  $\mathrm{Update}\;\mathrm{the}\;\mathrm{model}\;\mathrm{weights}\;\mathrm{using}\;{Loss}_{total}$ 20:  n ← n + 1 21: **End while**

### 3.6. Anomaly Criterion

For the anomaly scoring criteria, we also adopt a dual standard based on reconstruction deviation and latent space distance, combined with threshold setting.

Anomalous data points typically exhibit a larger distance in the latent space compared to normal data points, as each memory unit contains a prototype of normal data patterns. The latent space distance is represented as the sum of the distances between each variable and its corresponding memory unit. The final anomaly score is defined as follows:

(11) $$AnomalyScore\;\Phi \left(S\right)={\left.\langle Softmax\left(\frac{1}{N}\sum_{j=1}^{N}{\left\|z_{t}^{j}\;-\;p^{j}\right\|}_{2}^{2}\right)\;⨀\;\frac{1}{N\;T}\;{\left\|X^{\left(l\right)}-\;\hat{X^{\left(l\right)}}\right\|}_{2}^{2}\rangle \right|}_{l=1,\ldots ,L;\;t=1,\ldots ,T}$$

where ⊙ is the element-wise multiplication. $AnomalyScore\left(X\right)\in R^{L}$, the anomaly scoring function, scores the input sequence at each window $X^{\left(l\right)}$. Anomalous data points receive a higher score compared to typical data points.

The entire testing process is shown in Algorithm 2.
**Algorithm 2** ITMMG Testing Algorithm **Required:** Training data $X\in R^{T\times N}$; trained model parameters; block number *L_enc_*. 01: $Z^{0}$ = Embedding(*X*) 02: for l = 1 to *L_enc_*: 03:  Self-attention layer is applied on variate tokens. 04:  $Z^{l-1}$$\;=\;\mathrm{LayerNorm}\;(Z^{l-1}$$\;+\;\mathrm{Self-Attn}(Z^{l-1}$)) 05:  $Z^{l}$$\;=\;\mathrm{LayerNorm}\;(Z^{l-1}$$\;+\;\mathrm{Feed-Forward}(Z^{l-1}$)) 06: **End for** 07: $\mathrm{for}\;\mathrm{each}\;\mathrm{variable}\;\mathrm{in}\;Z^{l}$ **do**: 08:  Compute attention scores between 09:   $\hat{z}=Softmax(z\;p^{T})\cdot p$ 10: **End for** 11: $\hat{Z^{L_{enc}}}=concat(\left[Z^{L_{enc}},\tilde{Z^{l}}\right],dim=1)$ 12: $\hat{X}=f_{dec}(\hat{Z^{L_{enc}}})$ 13: Calculate Anomaly Score ϕ(*X*) 14: Return ϕ(X)

## 4. Experiment

### 4.1. Benchmark Dataset

We conducted experiments on four publicly available real-world datasets. A description of these datasets is provided below, and their characteristics are summarized in Table 1.

1. PSM (Pooled Server Metrics [37]): The PSM dataset, which comes from eBay server computers, is openly accessible. It has 25 characteristics that describe server machine metrics, including memory and CPU use.
2. SMAP (Soil Moisture Active Passive [38]): The SMAP dataset is used by NASA and contains soil samples and telemetry information obtained from the Mars rover. It consists of 25 features, primarily used for studying the spatiotemporal variations of soil moisture.
3. SWaT (Smart Water Treatment [39]): This dataset is collected from a real-world water treatment plant and contains sensor data with 51 dimensions, collected from continuously operating critical infrastructure systems.
4. NIPS-TS-SWAN (Server Computer): This is a publicly available comprehensive multivariate time series benchmark dataset, derived from the solar photospheric vector magnetogram images extracted from the Spaceweather HMI Active Region Patch series [40,41].

### 4.2. Evaluation Metrics

We use precision (P), recall (R), and F1 score (F1) to assess the model’s anomaly detection capabilities. In the following text, they are denoted as P, R, and F1, respectively. TP denotes true positives, FP denotes false positives, TN denotes true negatives, and FN denotes false negatives.

(12) $$Precision=\;\frac{TP}{TP+FP}$$

(13) $$Recall=\frac{TP}{TP+FN}$$

(14) $$F1=2\times \frac{Precision\times Recall}{Precision+Recall}$$

Higher values of these metrics indicate better detection performance by the model. The F1 score offers a more impartial assessment. Therefore, the F1 score is considered a more comprehensive metric.

Since anomaly samples are typically in the form of continuous anomalous segments, we adopt a point adjustment method. If a single timestep is identified as anomalous, it is presumed that all associated timesteps within the corresponding anomalous interval are likewise recognized accurately [42].

### 4.3. Implementation Details

Our experimental setup is summarized as follows. We employed non-overlapping sliding windows to extract subsequences. The sliding window size was fixed at 64, with 8 attention heads, 3 encoder layers, an embedding dimension of 512, and 10 memory units per variable. The temperature hyperparameter τ was set to 0.1, and the loss balancing coefficient λ was set to 0.01 to balance the two components of the loss function. All experiments were implemented using PyTorch 2.1.0 and optimized with the Adam [43] optimizer. The batch size was set to 32, the initial learning rate was configured to 10^−4^, and the experiments were conducted on a single NVIDIA GeForce RTX 3060Ti GPU (Colorful Technology Co., Ltd., Shenzhen, Guangdong, China).

### 4.4. Detection Results

Our model was compared extensively with 10 baseline methods, including classical methods: OC-SVM [44] and Isolation Forest [4]; density-based model: DAGMM [13]; clustering-based methods: ITAD [45] and THOC [46]; reconstruction-based models: LSTM-VAE [15], Anomaly Transformer [9], OmniAnomaly [16], BeatGAN [22]; autoregressive models: LSTM and VAR; and contrastive representation learning methods: DCdetector [47]. The evaluation of multivariate time series anomaly detection is shown in Table 2. Overall, ITMMG demonstrates performance comparable with that of SOTA methods, confirming its effectiveness.

Additionally, we assessed the NIPS-TS-SWAN dataset from Table 1, which, compared to the other three datasets, is more challenging due to a higher variety of anomaly types. As shown in Table 3, ITMMG’s performance metrics exceed the DCdetector method.

### 4.5. Ablation Studies

We conducted a series of ablation experiments to evaluate the effectiveness of the key components of the proposed model. By progressively removing or replacing different modules, we comprehensively evaluated the impact of each component on performance, as shown in Table 4.

When the Inverted Tokenization module was removed (w/o Inverted Tokenization), the model adopted a timestep-driven embedding method, which failed to capture the fine-grained relationships between variables using the multivariate attention mechanism. Similarly, when the multi-memory module was removed (w/o multi-memory), the model was unable to establish independent multi-memory gates for each variable. Instead, it used a shared global memory unit, causing the specific features of different variables to become mixed. Therefore, performance significantly decreased. In contrast, our model consistently achieved the highest performance across all datasets, demonstrating that the proposed approach effectively captures both temporal dependencies in time series and inter-variable dependencies. The model’s capacity to identify anomalous characteristics is improved by the multivariate memory gates for multivariate time series, which efficiently recognize the typical characteristics of several variables.

In addition, we explored the effectiveness of the model across various anomaly evaluation standards, including standalone reconstruction performance, standalone latent space deviation, and different combination methods for reconstruction performance and latent space deviation: multiplication and multiplication.

As shown in Table 5, the multiplicative combination achieves the highest overall performance, outperforming other fusion strategies across all evaluation metrics, as it enables better collaboration between reconstruction performance and latent space deviation. Numbers in bold represent the best-performing values.

(15) $$LSD\left(z,p\right)={\left.\langle Softmax\left(\frac{1}{N}\sum_{j=1}^{N}{\left\|z_{t}^{j}-p^{j}\right\|}_{2}^{2}\right)\;\rangle \right|}_{t=1,\ldots ,T}$$

(16) $$RDF\left(X\right)={\left[{\left\|X_{t,:}-{\hat{X}}_{t,:}\right\|}_{2}^{2}\right]}_{t=1,\ldots ,T}$$

(17) $$\mathrm{Latent}\;\mathrm{space}\;\mathrm{deviation}\;(\mathrm{LSD})\text{:}\;\phi \left(X\right)=LSD\left(z,\;p\right)$$

(18) $$\mathrm{Reconstruction}\;\mathrm{deviation}\;\mathrm{factor}\;(\mathrm{RDF})\text{:}\;\phi \left(X\right)=RDF\left(X\right)$$

(19) $$\mathrm{Addition}\text{:}\;\phi \left(X\right)=LSD\left(z,\;p\right)+RDF\left(X\right)$$

(20) $$\mathrm{Multiplication}\text{:}\;\phi \left(X\right)=LSD\left(z,\;p\right)\;⨀\;RDF\left(X\right)$$

### 4.6. Parameter Sensitivity

This section further explores how critical hyperparameter configurations influence the performance of ITMMG. These hyperparameters include window size L, embedding dimension size $d_{model}$, number of encoder layers, number of attention heads, and number of decoder layers. We conducted hyperparameter analysis experiments on three datasets as follows.

**Figure 7:**
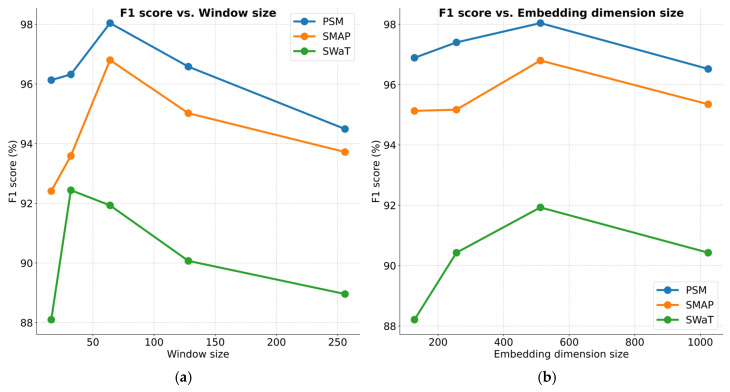

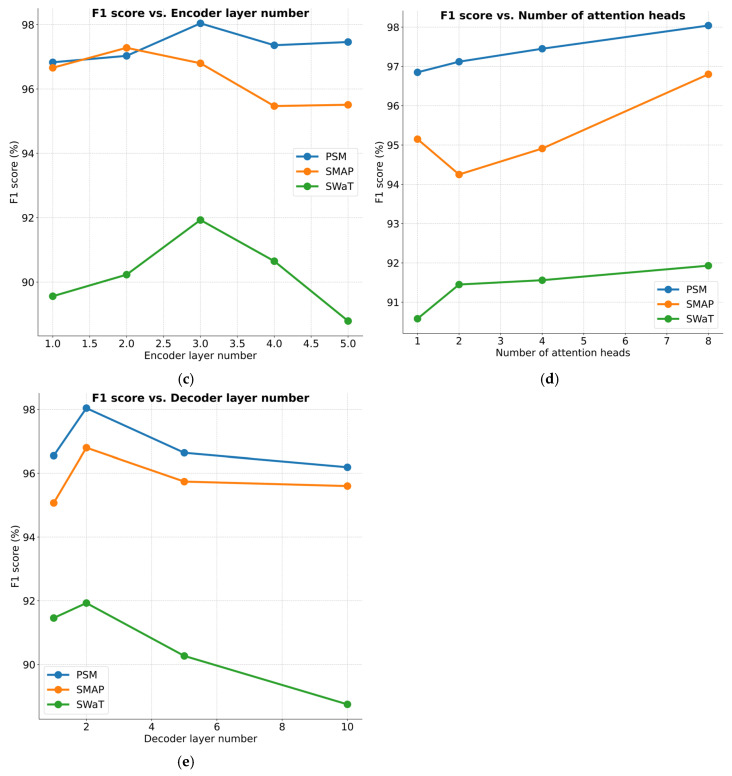
Parameter sensitivity studies of main hyper-parameters.

Figure 7a presents a comparative analysis of model effectiveness under varying window lengths. When the window size is smaller than 32, performance significantly drops. A small window size provides limited contextual time information, but within the range of window sizes from 32 to 256, the model shows robustness, with performance fluctuations of less than 2.31%. Figure 7b shows the performance results for different embedding dimension sizes. Overall, the effectiveness of the proposed method remains consistent across varying conditions. However, for the SWaT dataset, performance drops noticeably when the embedding dimension is reduced to 128. A very small embedding dimension may result in insufficient extraction of window timestep information, affecting the learning of representations in conjunction with the multivariate memory units. In the implementation, we used a window size of 64 to balance memory consumption and computational efficiency. Figure 7c,d illustrate the performance results across varying numbers of encoder layers and attention heads, respectively. Overall, the model’s performance remains stable. Figure 7e highlights the impact of different decoder layer numbers on performance. A smaller number of decoder layers leads to performance degradation due to insufficient reconstruction capability for input data, while an excessive number of layers increases the risk of over-generalization and reduces computational efficiency.

## 5. Conclusions

This paper proposes a multivariate time series anomaly detection method based on an inverted Transformer framework and a multi-memory gating mechanism. By employing an inverted token encoding strategy, the method encodes each variable in the time series independently, enabling finer-grained feature representations. This architectural choice enhances the model’s ability to model intricate variable-level relationships with greater precision. Meanwhile, the multi-memory gating mechanism assigns independent memory units to each variable, adaptively extracting normal patterns for different variables. This strengthens the model’s capability to detect outlier samples effectively. Empirical evaluations conducted on four publicly available datasets reveal that ITMMG surpasses many advanced methods in both detection precision and model robustness. Ablation experiments confirm the contribution of each individual module to the overall system performance.

Future research will focus on assessing the potential of ITMMG across various application domains, as well as enhancing its computational performance.

## Figures and Tables

**Table 1:** Dataset information.

Dataset	Dimension	Train	Validation	Test	Anomalies (%)
PSM	25	105,984	26,497	87,841	27.75
SMAP	25	108,146	27,037	427,617	13.13
SWaT	51	396,000	99,000	449,919	11.98
NIPS-TS-SWAN	38	48,000	12,000	708,420	32.6

**Table 2:** Overall results for real-world multivariate datasets. Precision (P), Recall (R), and F1 scores are used as evaluation metrics. All results are in %. For these three metrics, higher values indicate better performance, and the best ones are in bold.

Dataset	PSM			SMAP			SWaT		
Metric	P	R	F1	P	R	F1	P	R	F1
OC-SVM	62.75	80.89	70.67	53.85	59.07	56.34	45.39	49.22	47.23
IFOREST	76.09	92.45	83.48	52.39	52.39	55.53	49.29	44.95	47.02
DAGMM	93.49	70.03	80.08	86.45	56.73	68.51	89.92	57.84	70.4
VAR	90.71	83.82	87.17	81.38	53.88	64.83	81.59	60.29	69.34
LSTM-VAE	73.62	89.92	80.96	92.2	67.75	78.1	76.03	89.5	82.2
OmniAnomaly	88.39	74.46	80.83	92.49	81.99	86.92	81.42	84.3	82.83
BeatGAN	90.3	93.84	92.04	92.38	55.85	69.61	64.01	87.46	73.92
THOC	88.14	90.99	89.54	92.06	89.34	90.68	93.94	86.36	85.13
Anomaly Transformer	96.91	**98.9**	97.89	94.13	**99.4**	96.69	91.55	86.73	91.07
DCdetector	96.62	97.32	96.97	94.44	97.8	96.09	**92.53**	88.59	90.52
ITMMG (Ours)	**97.61**	98.47	**98.04**	**98.86**	94.82	**96.8**	92.38	**91.49**	**91.93**

**Table 3:** Overall results for NIPS-TS-SWAN. All results are in %, and the best ones are in bold.

Dataset	NIPS-TS-SWAN		
Metric	P	R	F1
OC-SVM	47.4	49.8	48.5
Iforest	56.9	59.8	58.3
MatrixProfile	16.7	17.5	17.1
GBRT	44.7	37.5	40.8
LSTM-RNN	52.7	22.1	31.2
Autoregression	42.1	35.4	38.5
AutoEncoder	49.7	52.2	50.9
AnomalyTransformer	90.7	47.4	62.3
DCdetector	95.5	**59.6**	73.4
ITMMG (Ours)	**99.32**	58.44	**73.58**

**Table 4:** Performance results of the ablation experiment. The best F1 score is in bold.

Method	F1 Score (%)				
	PSM	SMAP	SWaT	NIPS-TS-SWAN	Avg F1
w/o Inverted Tokenization	77.93	79.45	72.67	58.76	72.20
w/o multi-memory	81.57	80.26	82.68	59.57	76.02
w/o memory	83.21	81.23	73.23	60.12	74.44
ITMMG (Ours)	**98.04**	**96.8**	**91.93**	**73.58**	**90.09**

**Table 5:** Ablation of criterion definition.

Method	F1 Score (%)				
	PSM	SMAP	SWaT	NIPS-TS-SWAN	Avg F1
LSD	82.79	80.34	70.83	65.46	74.86
RDF	78.41	79.32	75.85	68.46	74.51
Addition	92.79	92.27	88.49	69.73	85.82
Multiplication	**98.04**	**96.8**	**91.93**	**73.58**	**90.09**

## Data Availability

The original contributions presented in this study are included in the article. Further inquiries can be directed to the corresponding author.
